# Dynamics and growth rate implications of ribosomes and mRNAs interaction in *E. coli*

**DOI:** 10.1016/j.heliyon.2022.e09820

**Published:** 2022-06-28

**Authors:** Tin Phan, Changhan He, Irakli Loladze, Clay Prater, Jim Elser, Yang Kuang

**Affiliations:** a School of Mathematical and Statistical Sciences, Arizona State University, Tempe, AZ 85287, USA; b Division of Theoretical Biology and Biophysics, Los Alamos National Laboratory, Los Alamos, NM 87544, USA; c Bryan Medical Center, Bryan College of Health Sciences, Lincoln, NE 68506, USA; d Department of Integrative Biology, Oklahoma State University, Stillwater, OK 74078, USA; e Flathead Lake Bio Station, University of Montana, Polson, MT 59860, USA

**Keywords:** Inactive ribosomes, Model validation, Growth rate hypothesis, Mathematical analysis, Cell growth, Translation dynamics, Model formulation

## Abstract

Understanding how cells grow and adapt under various nutrient conditions is pivotal in the study of biological stoichiometry. Recent studies provide empirical evidence that cells use multiple strategies to maintain an optimal protein production rate under different nutrient conditions. Mathematical models can provide a solid theoretical foundation that can explain experimental observations and generate testable hypotheses to further our understanding of the growth process. In this study, we generalize a modeling framework that centers on the translation process and study its asymptotic behaviors to validate algebraic manipulations involving the steady states. Using experimental results on the growth of *E. coli* under C-, N-, and P-limited environments, we simulate the expected quantitative measurements to show the feasibility of using the model to explain empirical evidence. Our results support the findings that cells employ multiple strategies to maintain a similar protein production rate across different nutrient limitations. Moreover, we find that the previous study underestimates the significance of certain biological rates, such as the binding rate of ribosomes to mRNA and the transition rate between different ribosomal stages. Furthermore, our simulation shows that the strategies used by cells under C- and P-limitations result in a faster overall growth dynamics than under N-limitation. In conclusion, the general modeling framework provides a valuable platform to study cell growth under different nutrient supply conditions, which also allows straightforward extensions to the coupling of transcription, translation, and energetics to deepen our understanding of the growth process.

## 1 Introduction

Growth is a fundamental process of life. The study of cell growth has always been of great interest to the scientific community, especially with increasing technological advances that allow for precise measurements and modifications of the biochemical composition and gene expressions of cells [1]. Protein synthesis is directly linked to cell growth rate, in particular during the exponential growth phase. However, the overall cell growth is a complex process that involves other heavily regulated cell functions that are necessary for cell survival [2], [3]. Thus, understanding how cells distribute their resources for growth and survival under different conditions is crucial to obtain a complete picture of integrated cellular function.

One promising direction examines optimal resource allocation theory to provide a quantitative framework to study cell growth under contrasting nutrient supply conditions [4], [5]. The theory proposes that cell growth is the result of optimal resource allocation, which is usually embedded in simple models of the translation process. Some of the earliest developments hypothesize the so-called “the constant-efficiency (of ribosome action) hypothesis”, which suggests that, since protein production is the most limiting process involved in cell growth, cell should optimize the protein synthesis rate of ribosomes to keep it at an optimal maximum regardless of situation. This implies that for one cell to grow twice as fast as another, it then must have twice the number of ribosomes [2], [6], [7]. This concept is an underlying principle behind “the growth rate hypothesis”, an important component in the study of ecological stoichiometry, which uses mass balance principles to link growth to organismal biochemistry and elemental composition [8], [9].

A version of the constant-efficiency hypothesis takes the form of a simple system of differential equations involving protein translation by ribosomes and self-replicating ribosomes [10]. An important implication of the model is that the protein to RNA ratio is linearly proportional to the growth rate, which is supported experimentally [4], [11]. While the translation rate may be relatively constant across different growth rates, it has been noted that there maybe alternative mechanisms that cells employ under different scenarios, especially during nutrient shifts or different types of nutrient limitation [6], [10], [11].

In a recent study, Li and colleagues carried out experiments to more deeply assess how the growth of *E. coli* is affected by carbon (C), nitrogen (N), and phosphorus (P) limitation. They find that *E. coli* uses three different mechanisms to maintain the same growth rate under the three scenarios [12]. In the same year, Iyer et al. (2018) reported how C and N starvation would affect cell growth and also find distinct patterns of transcription and translation regulation [13].

To qualitatively and quantitatively describe complex experimental findings, a theory or mathematical model is often necessary. One popular approach in *E. coli* studies [14] is to build a quantitative model that incorporates all relevant biological mechanisms and experimental parameters to describe the complex regulation system, as seen in the work by Hu et al. [15]. On the other hand, a simple model can also be built based on fundamental principles. One benefit of simpler models is that it can often be studied thoroughly to extract insights regarding general behaviors of the biological system, which can then be used in the formulation of more complex models. For example, a class of stochastic model called Totally Asymmetric Simple Exclusion Process (TASEP) which describes particles hopping on a one-dimension chain that exhibits a broad range of complex behaviors, has been used widely in biophysical literature to explore core elements of biological processes that involved transportation of biological matters such as translation [16], [17], [18], [19].

Additionally, Scott and colleagues constructed a simple model by arguing that cell growth must be balanced between varying levels of translation inhibition and varying nutrient quality [4]. Furthermore, Scott et al. also employed the approach of partitioning the ribosomes and protein into multiple factions each with its own function, which is further explored in subsequent studies [20]. Perhaps inspired by this idea, Li et al. constructed a model which partitions ribosomes and mRNA into several classes to describe the translation process under different nutrient limitations.

The model by Li et al. produces predictions that are consistent with experimental results to elucidate on biological mechanisms of cell growth and thus is of interest for further exploration. However, the study focuses on the experimental evidence, so the analytical properties and biological insights of the model are not explored in great detail. For example, it is unclear whether the model dynamics satisfy the assumptions used in its construction, or perhaps contain interesting dynamical properties. Furthermore, the authors use the steady state values for their calculations without guaranteeing their existence, uniqueness, positivity, and stability. In addition, to bypass the need for parameter estimations of two unknowns, the authors absorbed the two unknowns into a new parameter called the saturation parameter and use it to characterize the cell growth under different nutrient profiles. While ingenious from an analytical point of view, this approach does not allow for simulation of the dynamics of the model since the values and ranges of two parameters are unknown. Furthermore, the saturation parameter is somewhat ambiguous and unlikely to be experimentally measurable, reducing its usefulness to provide more in-depth biological insights into mechanisms regulating organismal growth.

Therefore, it is critical to accurately estimate the values of the biologically interpretable unknown parameters to obtain further hypothesis on contrasting effects of limitations by different nutrients on cell growth. In order to do so, we first generalize the model formulation by Li and colleagues and examine the interpretation and possible connection between each parameter. We then calibrate the model using the experimental results from Li et al. and discuss their implications on the growth dynamics and growth strategies under different nutrient limitation conditions. By thoroughly examining the model, we obtain valuable insights that may allow for better future model calibrations and developments to reach a more complete theory of cell growth.

## 2 Methods

In this section, we will first motivate and discuss the formulation of the macroscopic model by Li and colleagues and how it was used to describe the experimental data. Then, we will describe our approach to analyze the model and study its connections and implications using the experimental measurements by Li et al. [12].

### 2.1 Model motivation

Various formulations of a mathematical expression for cell growth rate exist [4], [5], [6], [10]. In the work of Li and colleagues, the growth rate was looked at from the perspective of protein mass accumulation in the translation process of each cell. Let $J_{p}$ be the protein synthesis rate, or the amount of proteins being synthesized per second (amino acids/sec), $P_{m}$ be the protein mass in a cell (g), and $m_{aa}$ be the average mass of amino acid (g). Then the amount of protein mass produced every second is the product of the protein synthesis rate and the average mass of amino acid ($J_{p}m_{aa})$. In other words, the growth rate, *μ* ($hr^{-1}$), is the relative increase in protein mass rate:

(1) $$\mu =\frac{3600J_{p}m_{aa}}{P_{m}},$$

where 3600 is the conversion factor from seconds to hour. Additionally, assuming the elongation rate in the translation process is a constant, then the protein synthesis rate (or the amount of amino acids being translated per second) is proportional to the number of ribosomes working on the translation process ($R_{w}$).

(2) $$J_{p}=R_{w}k_{el},$$

where $k_{el}$ is the elongation rate. Equating the protein synthesis rate in both equations and solve for the growth rate, we obtained an expression of the growth rate in term of the working ribosomes:

(3) $$\mu =R_{w}k_{el}\frac{3600m_{aa}}{P_{m}}.$$

Ideally, a model describing the growth of cells (in a chemostat) should produce a uniquely globally stable steady state. Thus, if we suppose the dynamics of cell growth indeed reaches a steady state, then the growth rate is proportional to the constant number of working ribosomes. Therefore, we want a model that can capture the dynamics of different fractions of ribosomes during the translation process to explain the impacts of different nutrient limitation conditions on growth rate.

### 2.2 Model formulation

To provide a theoretical framework for different mechanisms of cell growth under various nutrient limitations, Li and colleagues formulated the following kinetic ODEs describing the translation dynamics of ribosomes (R) and mRNA (M). The assumptions of the model will be marked in bolded texts with the superscript denoting the numerical order.

Ribosomes are separated into three groups. $R_{u}$ signifies the unbound ribosomes, which includes the free ribosomes and ribosomal sub-units 30S and 50S (i.e. separate, un-assembled sub-units are considered as part of $R_{u}$). $R_{i}$ is the initiating ribosomes, or the ribosomes that successfully bind to the initiation site and occupy no more than the first 10 codons (since one ribosome occupies about 10 codons on an mRNA). $R_{w}$ signifies the working ribosomes, which are bound to mRNA starting at the $11^{th}$ or higher numbered codons.

The bound ribosomes are composed of the initiating ribosomes and the working ribosomes. The differentiation between initiating and working ribosomes is supported using a microscopic model and some experimental data (see the supplementary material in [12]); however, this distinction, especially the length of mRNA corresponding to $R_{i}$, has not been explored in depth in literature, so it remains an ***assumption***^(1)^ of the model formulation. We will not discuss the microscopic model in detail here; however, the microscopic version resembles a continuous-differential version of the TASEP, and perhaps can be further analyzed within the existing framework of TASEP [18]. The main functional difference is that during the initiating phase, ribosomes can abort translation, while they cannot do so during the working phase.

The mRNA is categorized as free ($M_{f}$) and bound ($M_{b}$); however, a distinction should be noted because of a novel characterization of the mRNA. $M_{f}$ is used to indicate when the binding site of the mRNA is available for binding, and $M_{b}$ is used when the binding site is occupied by a ribosome $\left(R_{i}\right)$. In other words, $M_{f}(t)$ represents the number of mRNA with an available binding site (i.g. unbound at the first 10 codons), and $M_{b}$ is the number of mRNA whose binding site is currently occupied.

Note that the definition of $M_{f}$ is not exactly indicative of the starting region that includes the Shine-Dagalno sequence, but rather whether the mRNA is available for another ribosomes to bind to it or not. Li and colleagues argue that the binding region contains up to the first 10 codons of the mRNA (or the approximate occupancy of one ribosome on an mRNA). This classification allows the model to be structurally simple, while capturing key biological features of the process.

#### 2.2.1 General framework

The interactions between different components of the system can be characterized as follows. Unbound ribosomes can bind to free mRNA to become initiating ribosomes. We let $f(R_{u},M_{f})$ represent this binding action. In principle, the more abundant $R_{u}$ and $M_{f}$, the higher $f(R_{u},M_{f})$ should be, so *f* is a monotone function with respect to both variables. Furthermore, $f(0,0)=f(0,M_{f})=f(R_{u},0)=0$. For mathematical simplicity, *f* is also assumed to be differentiable everywhere except at (0,0). This interaction is represented below, where $f(R_{u},M_{f})$ is the functional form of the binding process. We note that the function form of $f(R_{u},M_{f})$ may contain parameters that relates to the binding rate of $R_{u}$ to $M_{f}$. Such parameters maybe under the influence of nutrient limitations.

(4) $$R_{u}+M_{f}\overset{f(R_{u},M_{f})}{\to }R_{i}+M_{b}.$$

Initiating ribosomes can abort translation, which revert to unbound ribosomes and free mRNA. Otherwise, the initiating ribosomes proceed to the 11th codon to become working ribosomes, which also releases the binding site, or increases the number of mRNA with available binding site. It is worth pointing out that the classification of mRNA allows the model to naturally incorporate the possibility that multiple ribosomes can translate a single strand of mRNA at the same time (e.g. polysomes). An alternative method to denote this multiple binding of ribosomes to mRNA is to use a chemical binding formulation, where the mass action approximation would be $f(R_{u},M_{f})=\alpha R_{u}^{n}M_{f}$, where *n* is the average number ribosomes translating one mRNA strand at a given time and *α* is a first-order binding rate. However, this process cannot account for the fact that ribosomes can only bind to mRNA one at a time. Another method is to use the TASEP framework as mentioned in the introduction; however, this method is perhaps more suitable if we want to accurately capture the microscopic nature of the translation process.

During the working phase, ribosomes do not abort translation and continue translating at a constant rate until the stop codon. We note that previous studies suggest that ribosomes can abort translation after the initiation phase [21], [22]. However, we elected to ignore this possibility in our model formulation and will discuss the implications of this choice in the discussion section. Upon arriving at the last coding codon, the working ribosomes complete the translation and unbind the mRNA to become free ribosomes (or ribosomal sub-units). In Li et al., the elongation rate is assumed to be independent of the progression rate from initiating to working ribosomes and the translation abortion rate. However, this creates several potential problems such as parameter unidentifiability and model interpretation. Instead, we note that the model readily provides a platform to connect these different transition rates.

We ***assume***^(2)^ that elongation rate $k_{el}(\cdot )$ is a function of the nutrient composition of the medium. Since the composition in each experiment is fixed, $k_{el}$ is taken to be a constant for each experiment. $k_{el}$ accounts for how many amino acids (*aa*) are added to the polypeptide chain per second (sec), so it has the unit (aa/sec). Thus, the amount of initiating ribosomes becoming working ribosomes per second is given by $k_{el}/M_{aa}$, where $M_{aa}$ is the length of mRNA corresponding to the initiating stage (e.g. $M_{aa}$ is taken to be the first 10 codons in Li et al.). Recall that this is possible because the section of the mRNA that determines its binding availability is not restricted to just the binding site. Similarly, $k_{el}/(N_{aa}-M_{aa})$ is the fraction of the coding mRNA region ($N_{aa}-M_{aa}$) translated by a working ribosome in one second, where $N_{aa}$ is the full length of the mRNA (i.g. from the 5' cap to the 3' end).

Regarding the translation aborting events during the initiating phase, we introduce a function g(⋅) which is the probability of aborting a translation. To simplify the biological mechanisms regarding g(⋅), such as the regulatory actions of guanosine pentaphosphate (p)ppGpp under nutrient limitation [23], we ***assume***^(3)^ g(⋅) to be dependent on the nutrient composition of the medium, so it also stays relatively constant within one experiment. Furthermore for consistency with the modeling framework, the aborting event is ***assumed***^(4)^ to be decided at the last coding codon of mRNA corresponding to $R_{i}$. It is worth noting that if the initiating region includes up to the first 10 codons (as in the original study), then the aborting event can only happen when the ribosome completely occupies the initiating region (prior to entering the working region) because a single ribosome occupies about 10 codons. Thus, the rate at which $R_{i}$ becomes $R_{w}$ is given by $\left(k_{el}/M_{aa})(1-g\right)$, which serves the original purpose of $k_{p}$. On the other hand, the rate at which $R_{i}$ aborts translation is given by $(k_{el}/M_{aa})g$, which serves the original purpose of $k_{r}$. Together, these processes can be represented as:

(5) $$R_{u}+M_{f}\overset{\frac{k_{el}}{M_{aa}}g(\cdot )}{\leftarrow }R_{i}\overset{\frac{k_{el}}{M_{aa}}(1-g(\cdot ))}{\to }R_{w}+M_{f},$$

(6) $$R_{w}\overset{\frac{k_{el}}{N_{aa}-M_{aa}}}{\to }R_{u}.$$

The differential equation system for this translation model takes the following form.

(7) $$\frac{dR_{u}}{dt}=\underset{\text{ribosomes released from aborted translation}}{\underset{︸}{\frac{k_{el}(\cdot )}{M_{aa}}g(\cdot )R_{i}}}+\underset{\text{ribosomes released from completing translation}}{\underset{︸}{\frac{k_{el}(\cdot )}{N_{aa}-M_{aa}}R_{w}}}-\underset{\text{ binding of ribosomes and mRNA}}{\underset{︸}{f(R_{u},M_{f})}},$$

(8) $$\frac{dR_{i}}{dt}=\underset{\text{ bound ribosomes from binding of ribosomes and mRNA}}{\underset{︸}{f(R_{u},M_{f})}}-\underset{\text{translating rate of ribosomes through the initiating phase}}{\underset{︸}{\frac{k_{el}(\cdot )}{M_{aa}}R_{i}}},$$

(9) $$\frac{dR_{w}}{dt}=\underset{\text{working ribosomes transitioned from initiating ribosomes}}{\underset{︸}{\frac{k_{el}(\cdot )}{M_{aa}}\left(1-g(\cdot )\right)R_{i}}}-\underset{\text{translating rate of ribosomes through the working phase}}{\underset{︸}{\frac{k_{el}(\cdot )}{N_{aa}-M_{aa}}R_{w}}},$$

(10) $$\frac{dM_{f}}{dt}=\underset{\text{binding site released from ribosomes leaving the initiating phase}}{\underset{︸}{\frac{k_{el}(\cdot )}{M_{aa}}R_{i}}}-\underset{\text{ binding of ribosomes and mRNA}}{\underset{︸}{f(R_{u},M_{f})}},$$

(11) $$\frac{dM_{b}}{dt}=\underset{\text{ bound mRNA from binding of ribosomes and free mRNA}}{\underset{︸}{f(R_{u},M_{f})}}-\underset{\text{binding site released from ribosomes leaving the initiating phase}}{\underset{︸}{\frac{k_{el}(\cdot )}{M_{aa}}R_{i}}}.$$

Note that the model does not contain any degradation or production terms, which is due mainly to the ***assumption***^(5)^ of time scale separation. The processes within translation happen on a much faster time scale (seconds) than the production and degradation of mRNAs and ribosomes (hours). Hence, degradation and production terms can be omitted. Furthermore, if we assume the total amount of ribosomes $R_{t}(\cdot )$ and the total amount of mRNA $M_{t}(\cdot )$ are constant for each experiment (perhaps using a quasi-steady state ***assumption***^(6)^ of the transcription process), then $R_{t}=R_{u}+R_{i}+R_{w}$ and $M_{t}=M_{f}+M_{b}$ with $R_{i}=M_{b}$ lead to a simplification of the full system to a two dimensional model.

(12) $$\frac{dR_{i}}{dt}=f(R_{t}-R_{i}-R_{w},M_{t}-R_{i})-\frac{k_{el}(\cdot )}{M_{aa}}R_{i},$$

(13) $$\frac{dR_{w}}{dt}=\frac{k_{el}(\cdot )}{M_{aa}}\left(1-g(\cdot )\right)R_{i}-\frac{k_{el}(\cdot )}{N_{aa}-M_{aa}}R_{w}.$$

It is worth pointing out that the dependence of $k_{el}(\cdot )$ and g(⋅) on nutrient composition only accounts for the nutrients specifically reserved or allocated for the translation process. Furthermore, without incorporating the transcription process, we assume the machinery needed for translation is at an optimal constant ($M_{t},R_{t}$). Regarding this observation, Li et al. attempted to study the effect of nutrient up-shift with an extended model; however, without explicitly incorporating the transcription process to account for temporal variation in the total number of ribosomes and mRNA, it may be misleading to extend their results to study the transient and asymptotic dynamics of cell growth after nutrient up-shift.

#### 2.2.2 Li et al. model formulation

In Li et al., the authors ***assumed***^(7)^ $f(R_{u},M_{f})$ takes the form of mass action. This assumption is justified as the number of ribosomes is much larger than the number of mRNA at almost any given time, especially at steady state growth. Furthermore, the effective rate at which ribosomes proceed to the 11th codon to become working ribosomes ($k_{p}$), the rate at which initiating ribosomes abort translation ($k_{r}$), and the elongation rate ($k_{el}$) are ***assumed***^(8)^ to be independent. We note a small inaccuracy regarding the term denoting the translation from the last coding codon to the stop codon, which should be the quotient between the elongation rate and the length of mRNA corresponding to working ribosomes (i.g., just the coding sequence on mRNA instead of the entire mRNA). However, since the length corresponding to the working ribosomes is nearly all of mRNA, we will use the original formulation, which does not affect the dynamical properties of the model.

For each nutrient limitation condition, $k_{el}(\cdot )$ is treated as constant (along with other parameters). Thus, under these assumptions, the model in Li et al. takes the following form.

(14) $$\frac{dR_{i}}{dt}=\underset{\text{binding of ribosomes and mRNA}}{\underset{︸}{k_{f}M_{f}R_{u}}}-\underset{\text{ initiating ribosomes aborting translation}}{\underset{︸}{k_{r}R_{i}}}-\underset{\text{initiating ribosomes becomes working ribosomes}}{\underset{︸}{k_{p}R_{i}}},$$

(15) $$\frac{dR_{w}}{dt}=\underset{\text{ working ribosomes from initiating ribosomes}}{\underset{︸}{k_{p}R_{i}}}-\underset{\text{translation from the last coding codon to the stop codon}}{\underset{︸}{\frac{k_{el}}{N_{aa}}R_{w}}},$$

(16) $$\frac{dM_{f}}{dt}=-\underset{\text{ binding of ribosomes and mRNA}}{\underset{︸}{k_{f}M_{f}R_{u}}}+\underset{\text{ aborted translation releases mRNA}}{\underset{︸}{k_{r}R_{i}}}+\underset{\text{producing working ribosomes releases mRNA}}{\underset{︸}{k_{p}R_{i}}}.$$

Using the conservation properties of the system, we can reduce equations (14)–(16) to two dimensional ODEs.

(17) $$\frac{dR_{i}}{dt}=k_{f}M_{t}R_{t}-k_{f}M_{t}R_{w}-(k_{f}M_{t}+k_{r}+k_{p}+k_{f}R_{t})R_{i}+k_{f}R_{i}^{2}-k_{f}R_{i}R_{w},$$

(18) $$\frac{dR_{w}}{dt}=k_{p}R_{i}-\frac{k_{el}}{N_{aa}}R_{w}.$$

**Figure 1:**
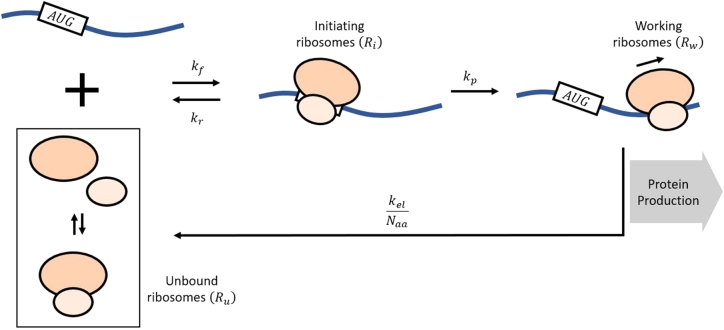
The schematics for ribosomal dynamics for Equations (14)–(16) adapted from Fig. 2b in the study by Li and colleagues [12]. Note that the classification for mRNA depends on whether the binding site is available for binding (*M*_*f*_) or not (*M*_*b*_).

A diagram of the system is presented in Fig. 1 and summaries of the model parameters and experimental values are presented in Tables 1 and 2, respectively. Recall the growth rate expression that we introduced in Equation (3). To obtain a more useful form, we first let $f_{r}$ and $m_{r}$ be the fraction of rRNA in RNA and the mass of rRNA in ribosomes, respectively. Then the total number of ribosome, $R_{t}$, is given by $R_{t}=P_{m}\xi \frac{f_{r}}{m_{r}}$, where *ξ* is the mass ratio of total RNA to total protein. Define $\Phi_{R_{w}}$ to be the fraction of working ribosomes (i.g. $\Phi_{R_{w}}=R_{w}/R_{t}$), then the new growth equation becomes:

(19) $$\mu =\xi \Phi_{R_{w}}k_{el}\left(3600m_{aa}\frac{f_{r}}{m_{r}}\right).$$

**Table 1:** Parameter ranges and definitions for *E. coli*. The ad hoc ranges are based on the estimated values in Li et al. [12]. The ranges indicate the min and max range for each parameter. During the parameter estimations, a more refined range is used for each scenario to obtain the best convergence. The model dynamic variables are listed at the end. The experimental variables and parameters are given in Table 2. The parameter indicated with ^⁎^ represents similar quantities with the parameters in the parentheses from Loladze and Elser [31].

Symbol	Definition	Ranges	Ref
*k*_*f*_	effective rate constant for unbound ribosomes and free mRNA to initiate translation (1/sec)	[1.0e − 5,2.4e − 4]	ad hoc
*k*_*r*_	rate constant for initiating ribosomes to abort translation (1/sec)	[5e − 3,5e − 2]	ad hoc
$k_{el}^{⁎}(\sigma_{a})$	elongation rate (*aa*/sec)	[6,20]	ad hoc
*k*_*p*_	rate constant for initiating ribosomes to transition into working ribosomes (1/sec)	[0.3,1.4]	ad hoc
*f*_*m*_	fractional mass of mRNA	0.02	[24]
*V*_*c*_	cell volume (m^3^)	10^−18^	[25]
*C*_*p*_	concentration of proteins (g/m^3^)	2.4 × 10^5^	[26]
*m*_*r*_	mass of rRNA component in a ribosome (g)	2.8 × 10^−18^	[27]
$m_{nuc}^{⁎}(m_{r})$	average mass of a nucleotide in RNA (g)	5.4 × 10^−22^	[28]
$m_{aa}^{⁎}(m_{a})$	average mass of an amino acid in protein (g)	1.8 × 10^−22^	[29]
*M*_*aa*_	average length of the initiating region (*aa*)	10	[30]
*N*_*aa*_	average length of mRNA (*aa*)	300	[30]
*n*_*a*_	N content averaged over 20 amino acids	17%	[31]
*n*_*r*_	N content averaged over 4 nucleotides	15%	[31]
*p*_*r*_	P content averaged over 4 nucleotides	9%	[31]
*l*_*a*_	length of RNA polymerase (aa)	3400	[31]
*l*_*r*_	length of rRNA (nt)	4560	[31]
*σ*_*r*_	transcription elongation rate (nt﹨sec)	71	[31]
*ϕ*_*r*_	fraction of total protein that is RNA polymerase actively transcribing rRNA	0.2%	[31]
*P*_*m*_	total protein mass in a cell (g)	*C*_*p*_*V*_*c*_	[12]
*R*_*t*_	total number of ribosomes per cell	$P_{m}\xi \frac{f_{r}}{m_{r}}$	[12]
*M*_*t*_	total number of mRNAs per cell	$P_{m}\xi \frac{f_{m}}{N_{aa}m_{nuc}3}$	[12]
*R*_*u*_	Number of Unbound ribosomes (count)		
*R*_*i*_	Number of initiating ribosomes (count)		
*R*_*w*_	Number of working ribosomes (count)		
*M*_*f*_	Number of mRNA with available binding site (count)		
*M*_*b*_	Number of mRNA with bound binding site (count)

**Table 2:** Summary of the mean of the processed experimental values for wild type *E. coli* from Li et al. [12]. The values of *f*_*r*_ are used as input and the values of *k*_*el*_ are used to cross check with our independent estimation of *k*_*el*_. The parameter indicated with ^⁎^ represents similar quantities with the parameters in the parentheses from Loladze and Elser [31].

Symbol	Definition	C-limit	N-limit	P-limit
*ξ*	Mass ratio of total RNA to total protein	0.16	0.16	0.08
*μ*	steady-state growth rate (h^−1^)	0.09	0.09	0.09
$\Phi_{R_{u}}$	Fraction of ribosomes not bound to mRNA	0.69	0.49	0.35
$\Phi_{R_{i}}$	Fraction of ribosomes located at the first 10 codons of mRNA that may abort translation prematurely	0.03	0.03	0.03
$\Phi_{R_{w}}^{⁎}(\phi_{a})$	Fraction of ribosomes located after the first 10 codons of mRNA, which contributes to the protein production	0.29	0.47	0.62
*f*_*r*_	Fractional mass of rRNA among total RNA	0.64	0.59	0.51
*k*_*el*_	Elongation rate (aa/sec)	12.5	7.5	12.5

Li and colleagues carefully parametrize the models with global constants and their experimental measurements [12]. In addition to the estimable parameters, the values of $k_{f}$ and $k_{r}$ are unknown. Instead, a new parameter called the saturation parameter (*S*) was introduced. As *S* is the only unknown, the value of *S* can be approximated. Finally, *S* was used to characterize the difference of cell growth under different nutrient limitation conditions.

Regarding cell growth under various nutrient limitations, there are three main findings by Li and colleagues corresponding with three different mechanisms under C-, N-, or P-limitation.

1. Under C-limitation, there is a low fraction of working ribosomes, which results in lower translation rate. However, the pool of ribosomes and the elongation rate are both high.
2. Under N-limitation, the elongation rate is slowed (due to stalling at glutamine codons) leading to a slow translation. However, the fraction of working ribosomes is moderate and a large pool of ribosomes compensates for the slow elongation.
3. Under P-limitation, there is a low abundance of ribosomes, which slows down translation. However, the lack of ribosomes is made up for by the high fraction of working ribosomes and fast elongation rate.

Their experimental results provide estimates of the fractions of free, initiating, and working ribosomes, i.g. $\Phi_{R_{u}},\Phi_{R_{i}},\Phi_{R_{w}}$ respectively, the elongation rates, and the abundance of ribosomes ($R_{t}$) calculated via the mass ratio of total RNA to total protein.

### 2.3 Parameter estimation

The main objective of our parameter estimation is to estimate possible values of $k_{r}$ and $k_{f}$ using the experimental values for $\mu ,\Phi_{R_{u}},\Phi_{R_{i}}$, $\Phi_{R_{w}}$ (at steady state), and *ξ* in each condition (C-limited, N-limited, and P-limited). Table 2 contains the experimental values from Li et al. [12]. Table 1 contains the ranges and fixed values of all parameters. Since one of our goals is to quantify/verify the difference in elongation rate under different nutrient-limited conditions, we will also estimate $k_{el}$, which means we will need to re-estimate $k_{p}$ (since $k_{p}$ is calculated at steady-state based on $k_{el}$).

**Data:** Li and colleagues performed various experiments using batch and continuous (chemostat) cultures, where the experiments using batch cultures were mainly for supporting findings from the continuous cultures. For our purpose, we will use the three sets of data for the *E. coli* strain NCM3722 at growth rate of 0.09 ${\text{hr}}^{-1}$ under three different nutrient limitations. In each experiment involving the wild type *E. coli*, Li and colleagues determine the mass ratio of total RNA to total protein and quantify rRNA and ribosome fractions. The elongation rate was also measured using lacZ induction; since the values for $k_{el}$ are from Fig. 6.a in the supplementary materials of Li et al., we approximate their values from that figure. The means of the processed experimental data are summarized in Table 2. Note that, in our parameter estimation, we will try to estimate the elongation rate $k_{el}$ directly and independently, so we will use their experimentally measured value to cross check. The values for $f_{r}$ are used as input for the model. For specific details of the experiments, please refer to the method section in Li et al. [12].

Our approach is similar to that of an Approximate Bayesian computation scheme, for additional details see [32]. Our goal is not to obtain a precise estimation of the posterior distribution, but rather to find admissible estimations that satisfy all biological measurements at the same time. Let $\theta =\{k_{f},k_{r},k_{el},k_{p},\xi \}$ and $x=\{\mu ,\Phi_{R_{u}},\Phi_{R_{i}},\Phi_{R_{w}}\}$.

1. First, we created $5\times 10^{5}$ samples $\theta^{⁎}$ from some uniform distributions π(θ).
2. Then, we simulated $5\times 10^{5}$ data set $x^{⁎}$ corresponding to $\theta^{⁎}$.
3. Finally, we compared $x^{⁎}$ with the experimental data $x^{0}$ from Li et al. [12]. In this case, we wanted each component of $x^{⁎}$ to be within 10% error of the corresponding element in $x_{0}$. Thus, we accepted the proposed parameter sets if
  (20) $$d(x_{i}^{0},x_{i}^{⁎})\leq 0.1⁎x_{i}^{0},$$
  where i=1,2,3,4 representing $\left\{\mu ,\Phi_{R_{u}},\Phi_{R_{i}},\Phi_{R_{w}}\right\}$, respectively, and d(⋅,⋅) is a measure of the absolute distance between its arguments.

To determine the range of π(θ) in step 1, we started with the baseline estimates from Li et al. whenever possible. If the range of certain parameters was not available, we were able to obtain an initial estimate using the upper and lower bounds of other parameters in combination with experimental estimated values of x at steady state. We slowly expand the range, about 10–20% each time, within biologically reasonable values until the fitting found no acceptable values near the lower and upper bounds.

To test for the possibility that a completely different set of parameters might also satisfy the acceptable criteria, we also expanded the lower and upper bounds by many-fold (10 to 100) with the exception of parameters that were well-constrained biologically (e.g., $k_{el}$ should not be too close to 0 or much larger than 20, etc.). While more systematic approaches exist in literature, the stable convergence in our results demonstrates the sufficiency of our method in estimating the parameter distributions.

### 2.4 Global sensitivity analysis

To study the influence of parameters under each nutrient profile and in general, we calculate the partial rank correlation coefficients (PRCC) using 2000 samples obtained from the Latin hypercube Sampling. The details of the scheme can be found in the studies by [33], [34]. We vary the parameters within 1% range assuming uniform distribution. As a separate scenario, we also vary the parameters within a 25% range to study the impact of more uncertainty in the parameter values. The sensitivity and uncertainty analyses are carried out for each nutrient limitation using the estimated and fixed parameters.

## 3 Results

### 3.1 Mathematical analysis

Recall that in the study by Li and colleagues, the focus was to establish a mathematical model that can explain the experimental observations. Thus, certain assumptions were made during the model formulation; however, whether the dynamical behaviors of the model satisfy those assumptions was not considered. Furthermore, the existence of a unique globally asymptotically stable positive steady state was assumed and used as part of several key algebraic manipulations. For these reasons, we aimed to verify the basic biological assumptions and the asymptotic behaviors of the model.

**Figure 2:**
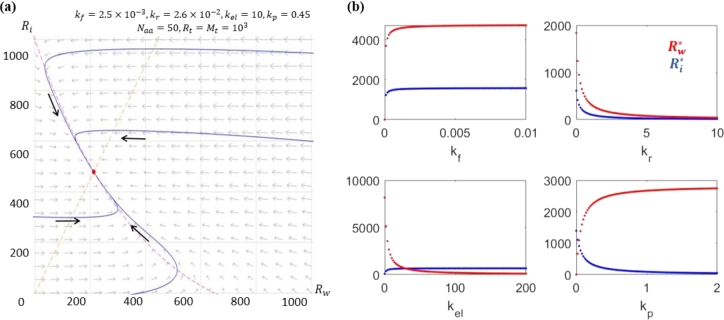
An example to demonstrate that the unique positive steady state is globally asymptotically stable. (a) Phase-plane with trajectory – note that the positive steady state exists within the biological bounds of *R*_*t*_. (b) Effects of varying model parameters on the asymptotic behaviors of the model. While the stability does not change, the relative ratios between $R_{w}^{⁎}$ and $R_{i}^{⁎}$ can be altered by changing *k*_*p*_ or *k*_*el*_. This is consistent with the steady state expression from Theorem 3, $R_{w}^{⁎}=N_{aa}\frac{k_{p}}{k_{el}}R_{i}^{⁎}$.

Theorem 1, Theorem 2 establish that each variable is always positive and bounded by the biological bounds ($M_{t}$ and $R_{t}$). Theorem 3 shows that the system always exhibits a unique biologically relevant positive steady state. Finally, Theorem 4 shows the local asymptotic stability of the positive steady state, which is indeed globally asymptotically stable by Theorem 5. Fig. 2 shows an example that demonstrates the stability of the system. These stability results provide mathematical justification for the algebraic manipulations that were done in Li et al. [12]. The proofs are given in subsection A.1 of the Appendix A.

Theorem 1 **Positivity**

*The system is positive given positive initials.*

Theorem 2 **Boundedness**

*Each compartment of ribosome is bounded by* $R_{t}$ *and each compartment of mRNA is bounded by* $M_{t}$ *.*

Remark 1 We note that the positivity and boundedness of the general model also hold similarly.

Theorem 3 **Positive equilibrium**

*With the biological bounds of* $R_{t}$ *and* $M_{t}$ *, then there is a unique positive equilibrium at* $\left(R_{i^{-}}^{⁎},R_{w^{-}}^{⁎}\right)$ *, where* $R_{i^{-}}^{⁎}=\frac{1}{A}\left(-B-\sqrt{B^{2}-4AC}\right)$ *and* $R_{w^{-}}^{⁎}=N_{aa}\frac{k_{p}}{k_{el}}R_{i^{-}}^{⁎}$ *with* $A=k_{f}\left(1-N_{aa}\frac{k_{p}}{k_{el}}\right),C=k_{f}M_{t}R_{t}$ *, and*

$$B=-\left(k_{f}M_{t}N_{aa}\frac{k_{p}}{k_{el}}+k_{f}M_{t}+k_{r}+k_{p}+k_{f}R_{t}\right).$$

Theorem 4 **Local stability**

*The biological equilibrium at* $\left(R_{i^{-}}^{⁎},R_{w^{-}}^{⁎}\right)$ *is locally asymptotically stable.*

Theorem 5 **Global stability**

*The biological equilibrium at* $\left(R_{i^{-}}^{⁎},R_{w^{-}}^{⁎}\right)$ *is globally asymptotically stable in the region bounded by*

$$C=\{R_{i},R_{w}>0;R_{i}+R_{w}\leq R_{t};R_{i}\leq M_{t}\}.$$

### 3.2 Computational results

**Figure 3:**
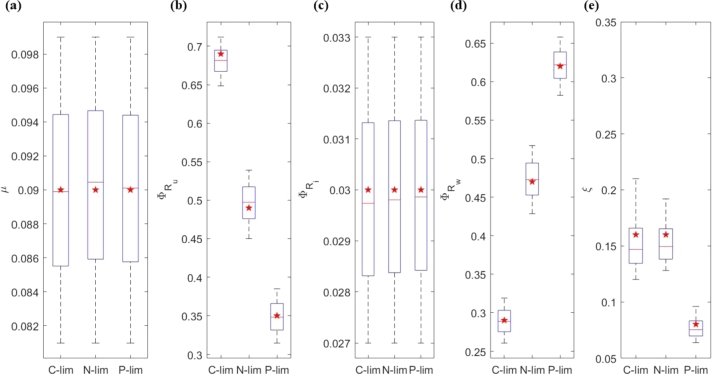
Model vs. experiments – the red star is the estimated value from the experiments. The red line is the median of the parameter estimation. The box represents the 25%–75% quantiles. The variables used for fitting are for: (a) *μ*, (b) $\Phi_{R_{u}}$, (c) $\Phi_{R_{i}}$, (d) $\Phi_{R_{w}}$, (e) *ξ*. The stars staying within the box means the simulation results agree well with the experimental results. The resulting parameter estimates are given in Table 3 and Fig. 4.

**Figure 4:**
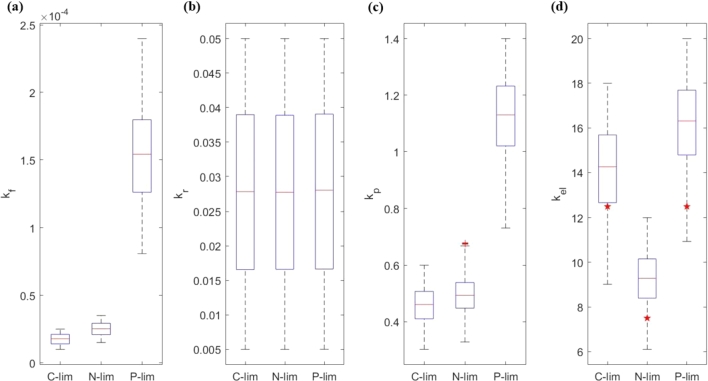
Parameter estimation from data fitting in Fig. 3 – the red star is the estimated value from the experiments (for the elongation rate only). The red line is the median of the parameter estimation. The box represents the 25%–75% quantiles. The red crosses are the outliers. The estimated parameters are: (a) *k*_*f*_, (b) *k*_*r*_, (c) *k*_*p*_, (d) *k*_*el*_.

Recall that our parameter estimation process focuses on finding sets of parameters that allow the model to fully capture the experimental values within an acceptable error range. Table 3 gives the estimated range of the parameters ($k_{r}$, $k_{f}$, $k_{el}$, $k_{p}$). Important simulation results are shown in Figs. 3 and 4. Multiple rounds of simulations confirmed that there were no significant differences in the convergence of the estimated parameters, see Figure 7, Figure 8 in Appendix subsection A.2

**Table 3:** Parameter estimations for the purpose to provide consistency between the model prediction and experimental results. The tables show the mean and standard deviation of the parameter values. The median and 25–75% quartiles are shown in Fig. 4 with the corresponding fitting in Fig. 3.

Params	C-lim	C-sd	N-lim	N-sd	P-lim	P-sd
*k*_*f*_	1.76 × 10^−5^	4.14 × 10^−6^	2.51 × 10^−5^	5.15 × 10^−6^	1.54 × 10^−4^	3.39 × 10^−5^
*k*_*r*_	2.77 × 10^−2^	1.29 × 10^−2^	2.77 × 10^−2^	1.29 × 10^−2^	2.79 × 10^−4^	1.30 × 10^−2^
*k*_*el*_	1.42 × 10^1^	1.97 × 10^0^	9.28 × 10^0^	1.20 × 10^0^	1.62 × 10^1^	1.90 × 10^0^
*k*_*p*_	4.59 × 10^−1^	6.43 × 10^−2^	4.94 × 10^−1^	6.23 × 10^−2^	1.12 × 10^0^	1.40 × 10^−1^

**Figure 5:**
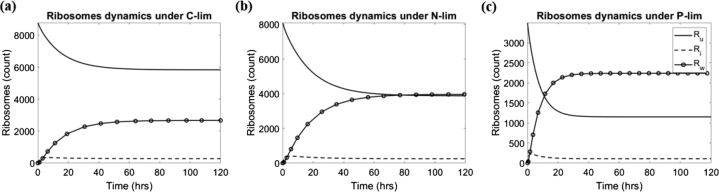
Ribosomes dynamics under different nutrient limitation conditions – simulated using the mean values from the parameter estimations presented in Table 3. The simulations are for: (a) C-limitation, (b) N-limitation, (c) P-limitation.

Using the estimated parameters in each case, we simulate the dynamics of ribosomes under C-, N-, and P-limitation, see Fig. 5. For the simulation, we assume that initially all ribosomes are unbounded, meaning $R_{u}(0)=R_{t}$. We observe that in all three scenarios, the dynamics of $R_{u}$ and $R_{w}$ are monotonically decreasing and increasing, respectively. $R_{i}$ reaches an early peak within the first few hours (1.71 hr under C-limitation, 5.14 hr under N-limitation, and 2.13 hr under P-limitation) before settling into a steady state. The steady state of $R_{i}$ is reached (within 10%) at 24.75 hr under C-limitation, 41.61 hr under N-limitation, and 22.17 hr under P-limitation. Additionally, under C-limitation, the ribosomal dynamics take 24.75 hr and 37.59 hr to reach within 10% of the equilibrium for $R_{u}$ and $R_{w}$, respectively. Under N-limitation, the ribosomal dynamics take 41.61 hr and 42.91 hr to reach within 10% of the equilibrium for $R_{u}$ and $R_{w}$, respectively. And under P-limitation, the ribosomal dynamics take 21.56 hr and 17.40 hr to reach within 10% of the equilibrium for $R_{u}$ and $R_{w}$, respectively.

We note that, while it is non-optimal to use the same set of initial conditions for the comparisons in Fig. 5, our main goal was to compare the rate at which the ribosome profile settles to the steady states under each nutrient limitation. Thus, by starting with the same initial values, the combined effect of all parameters dictates the reaction rate, allowing us to make relative comparisons across treatment. We also tested the ribosomal dynamics under four different scenarios, while there are qualitative differences, the ribosomal dynamics under C- and P-limitation is still faster than under N-limitation, see Fig. 9 in Appendix subsection A.2.

**Figure 6:**
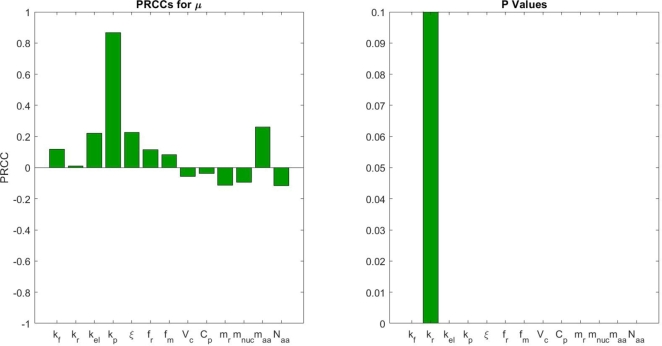
Global sensitivity with respect to the growth rate *μ* given large uncertainty in parameter values. The results indicate that *k*_*p*_ is the most sensitive parameter, while *k*_*r*_ is the least sensitive parameter. Additionally, the elongation rate *k*_*el*_ is among the most sensitive parameters, but fails behind *k*_*p*_. We suspect this is an artifact of the model formulation where *k*_*el*_ is assumed to be independent of *k*_*p*_.

**Figure 7:**
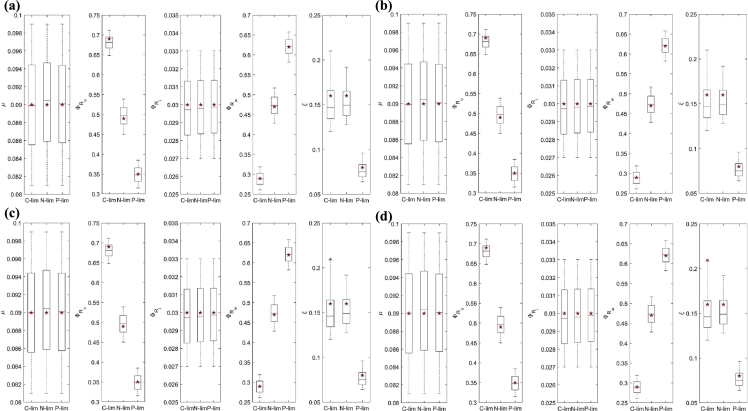
Testing the simulation convergence in Fig. 3 under four different scenarios: (a) *R*_*u*_ = 0.9*R*_*t*_,*R*_*i*_ = 0.05*R*_*t*_,*R*_*w*_ = 0.05*R*_*t*_; (b) *R*_*u*_ = 0.8*R*_*t*_,*R*_*i*_ = 0.10*R*_*t*_,*R*_*w*_ = 0.10*R*_*t*_; (c) *R*_*u*_ = 0,*R*_*i*_ = *R*_*t*_,*R*_*w*_ = 0.0; (d) *R*_*u*_ = 0,*R*_*i*_ = 0,*R*_*w*_ = *R*_*t*_. The convergence results are stable and agree with the result in Fig. 3 in all cases.

**Figure 8:**
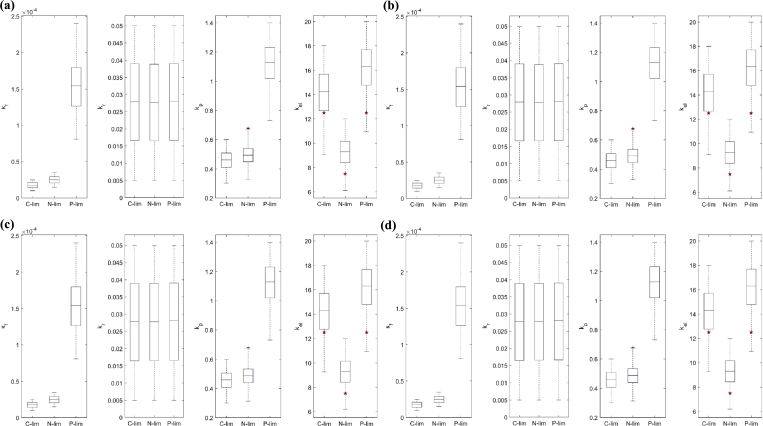
Testing the simulation convergence in Fig. 4 under four different scenarios: (a) *R*_*u*_ = 0.9*R*_*t*_,*R*_*i*_ = 0.05*R*_*t*_,*R*_*w*_ = 0.05*R*_*t*_; (b) *R*_*u*_ = 0.8*R*_*t*_,*R*_*i*_ = 0.10*R*_*t*_,*R*_*w*_ = 0.10*R*_*t*_; (c) *R*_*u*_ = 0,*R*_*i*_ = *R*_*t*_,*R*_*w*_ = 0.0; (d) *R*_*u*_ = 0,*R*_*i*_ = 0,*R*_*w*_ = *R*_*t*_. The convergence results are stable and agree with the result in Fig. 4 in all cases.

**Figure 9:**
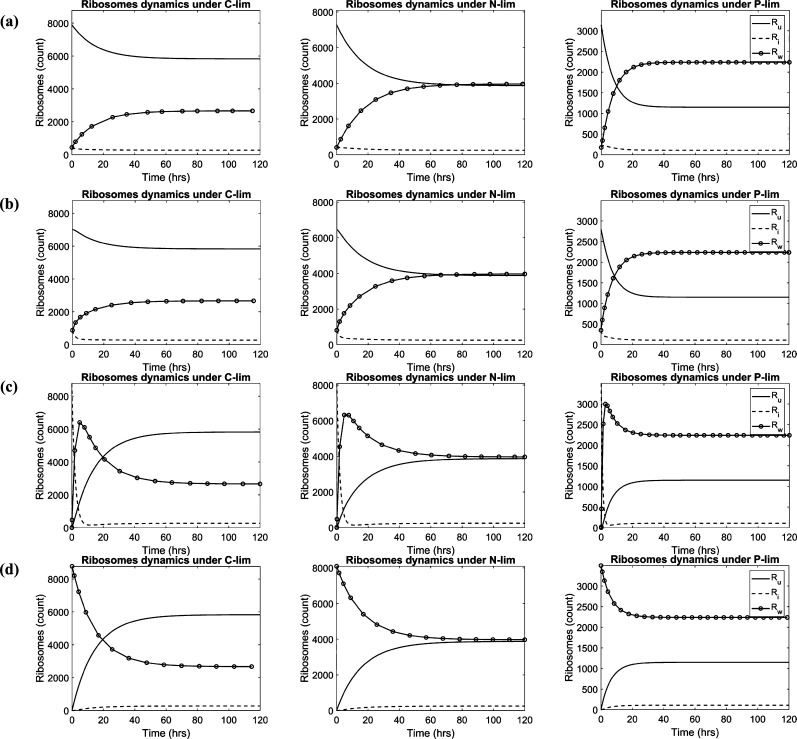
Testing the ribosomal dynamics, as compared to Fig. 5, with four different initial conditions: (a) *R*_*u*_ = 0.9*R*_*t*_,*R*_*i*_ = 0.05*R*_*t*_,*R*_*w*_ = 0.05*R*_*t*_; (b) *R*_*u*_ = 0.8*R*_*t*_,*R*_*i*_ = 0.10*R*_*t*_,*R*_*w*_ = 0.10*R*_*t*_; (c) *R*_*u*_ = 0,*R*_*i*_ = *R*_*t*_,*R*_*w*_ = 0.0; (d) *R*_*u*_ = 0,*R*_*i*_ = 0,*R*_*w*_ = *R*_*t*_. While there are qualitative differences in the dynamical behaviors (especially in the third and fourth scenarios), under C- and P-limitation, the convergence rate is faster compared to the situation under N-limitation.

Fig. 6 shows the global sensitivity results with respect to *μ* for large uncertainty in the parameters (10–200%). The result is with respect to the growth rate under C limitation; however, similar results are obtained for N- and P-limited conditions. We also carried out sensitivity analysis when the uncertainty is small (1%). Under both assumptions about the uncertainty level, we observe that *μ* is sensitive to all parameters with the exception of $k_{r}$ (all results are statistically significant except for $k_{r}$). Moreover, we note that $k_{p}$ seems to be the most impactful parameter. The sensitivity analysis is consistent with what we observe in our parameter estimation, where the values and ranges of $k_{r}$ are similar across all three nutrient limitations, see Fig. 4. This makes sense from our generalized derivations, where there is a hidden relationship between $k_{r},k_{p}$, and $k_{el}$. This suggests that least one of the three parameters is not identifiable, a recurring problem in mathematical modeling of biological systems [35].

### 3.3 Connection to the growth rate hypothesis

The growth rate hypothesis relates the abundance of P-rich ribosomal RNA to differences in maximal growth rate. But when the maximal growth rate is similar under different nutrient limitation conditions, it is of interest to study the N:P ratio of cell under each condition. Switching from considering Protein:RNA to N:P helps to identify the stoichiometric mass balance aspect of growth-limitation and gives a first-principle mechanistic understanding as to how organisms alter their elemental allocation to optimize growth. It also gives us simple predictions that can be tested empirically, so we can work iteratively to better understand nutritional controls on organismal biology.

Using the framework established by Loladze and Elser [31], we shall estimate the N:P ratio of the E.coli in the three nutrient limitation conditions in the study by Li and colleagues. Let *θ* denote the N:P mass ratio of the system. Note that the N content within a cell can be approximated by (N content average over 20 amino acids) × (mass of all protein) + (N content average over 4 nucleotides) × (mass of all rRNA). And the P content within a cell can be approximated by (P content average over 4 nucleotides) × (mass of all rRNA). Recall that $P_{m}$ is the mass of all proteins inside the cells. Let *r* be the mass of all rRNA; then the N:P mass ratio can be expressed as

(21) $$\theta =\underset{\text{N content}}{\underset{︸}{\left(n_{a}P_{m}+n_{r}r\right)}}:\underset{\text{P content}}{\underset{︸}{\left(p_{r}r\right)}},$$

where $n_{a}$ and $n_{p}$ represent the N content average over 20 amino acids and 4 nucleotides, respectively, while $p_{r}$ represents the P content average over 4 nucleotides. Loladze and Elser estimated that $n_{a}$ is between 15% to 18%, $n_{r}$ is approximately 15%, and $p_{r}$ is about 9%, so we take $n_{a}$, $n_{r}$, and $p_{r}$ to be 17%, 15%, and 9%, respectively, for our calculations. By using a model that couples protein and rRNA synthesis, Loladze and Elser reformed the above expression into the equivalence:

(22) $$\theta =n_{a}\frac{m_{a}}{m_{r}}\sqrt{\frac{l_{a}\phi_{a}\sigma_{a}}{l_{r}\phi_{r}\sigma_{r}}}+n_{r}:p_{r}.$$

The definitions of the parameters are recorded in Tables 1 and 2. Here, we note that $\phi_{a}$ and $\sigma_{a}$ are the same as $\Phi_{R_{w}}$ and $k_{el}$ in the Li et al. model. Furthermore, the $m_{a}$ and $m_{r}$ also represent similar quantities in both models. The bridge between the two models is the unique, globally, asymptotically stable equilibrium that allows us to pass the estimations from one model to the other. Thus, we can first estimate the values of $\Phi_{w_{k}}$ and $k_{el}$ with the Li et al. model, then use them to estimate the N:P mass ratio in each limitation condition, which then gives us the actual N:P ratio via a conversion based on their atomic numbers. The results are presented in Table 4.

**Table 4:** Estimation of N:P ratio under each nutrient limitation condition. The values are converted from *θ* using atomic ratio of N:P (14.007u:30.97376u). The first three estimates (without ^⁎^) used the experimentally reported values in Li et al. The second three estimates (with ^⁎^) used the numerically fitted values.

Condition	C-lim	N-lim	P-lim	C-lim^⁎^	N-lim^⁎^	P-lim^⁎^
N:P	9.76	9.68	12.57	10.16^⁎^	10.35^⁎^	13.80^⁎^

## 4 Discussion

The effects of nutrient conditions on cell growth have attracted significant modeling effort due to their wide implications, such as in the fields of ecology [36], [37], [38], [39], [40] and agriculture [41], [42], [43], [44], [45]. Furthermore, these effects introduce interesting dynamical properties extending the Lotka-Volterra framework [36], [37], [38], [46], [47]. While the overall aim is to reach a deeper understanding of biological systems by means of constructing a quantitative framework, the mathematical and biological properties of the model are often neglected. Without careful consideration, models may violate fundamental principles, such as mass conservation, or give unrealistic implications about the biological systems that they are meant to describe. In this study, we analyzed the model that Li et al. [12] formulated to describe their experimental data. Our main findings are summarized below.

**A general framework of *E. coli* translation process.** Mathematical models can be used to capture the essences of highly complex and nonlinear dynamic processes in a biological system. However, to do so, certain assumptions and generalization must be made. Thus, building upon the model formulation by Li and colleagues, we created a simple and generalizable mathematical framework for the translation process of cells such as *E. coli*. We stated all major assumptions related to the model formulation and pointed out possible limitations, especially with regards to the lack of the transcription process. Furthermore, when certain parameters are kept independent of one another, our theoretical and simulation results suggest a possible issue with parameter identification and interpretation.

**Existence, uniqueness, and stability of the positive steady state.** We analyzed the mathematical properties of the model developed by Li and colleagues to demonstrate that it satisfies the basic biological assumptions such as positive invariance and boundedness, specifically by the biological bounds established by the total number of ribosomes ($R_{t}$) and the total number of mRNA ($M_{t}$) from the model formulation. Furthermore, we confirm the existence of a unique positive steady state under all possible ranges of the parameter values, which is also globally asymptotically stable. This validates the algebraic manipulations based on the steady state values in Li et al. [12].

**Model consistency with experimental results.** Our results show that the model can produce simulation results that agree well with experimental results under all three scenarios of C-, N-, and P-limitations. This supports the usefulness of the framework in aiding our understanding of the nonlinear relationships between growth, biochemical and stoichiometric under differential nutrient limitation. Furthermore, we were able to estimate the values of the effective rate constant for unbound ribosomes and free mRNA to initiate translation ($k_{f}$) and the rate constant for initiating ribosomes to abort translation ($k_{r}$), which are previously unknown due to grouping into the saturation parameter *S*. While $k_{r}$ stays relatively constant across three nutrient regimes (perhaps due to identifiability issue), we observe significant variation in the estimated values of $k_{f}$ between P-limited vs. C- and N-limited environments. Even when transcription and energetics are not explicitly incorporated, the model is able to capture the observed experimental differences under three different nutrient profiles.

**Under P-limitation, the high fraction of working ribosomes and fast elongation rate are not sufficient to account for the lack of total ribosomes.** Assuming the rate that unbound ribosomes and free mRNA initiate translation, $k_{f}$, is similar across three nutrient conditions, we were unable to obtain simulations consistent with the experimental results in Li et al. Instead, by letting $k_{f}$ vary within an extended range (Table 1), we were able to simulate the experimental results. Specifically, we find that, under P-limitation, $k_{f}$ is several times larger than under C- or N-limitation, see Table 3. This implies that, other than having a higher fraction of working ribosomes and fast elongation rate, P-limited cells may also require a fast transition rate from initiating to working ribosomes to achieve the optimal growth rate. Additionally, our estimate of $k_{p}$ (transition rate from initiating ribosomes to working ribosomes) is also significantly higher under P-limitation, while remaining relatively similar under C- and N-limitation, see Fig. 4(c). These estimations suggest that, in order to overcome the lack of total ribosomes, multiple processes must work more rapidly. We suspect that this finding is mainly a consequence of lower value of *ξ* under P-limitation (see Table 2), which leads to a much lower amount of total ribosomes. For instance, a possible explanatory hypothesis is that suppose a similar amount of energy is reserved for translation across three different nutrient limitations. When there are less working components, it may appear that each component is working faster or more efficient due to a higher per capita energy allocation.

**The transition rate from initiating to working ribosomes is the most important factor regardless of which nutrient is limiting.** The results of our sensitivity analysis demonstrate that all parameters are sensitive with respect to the growth rate, with the most sensitive parameter being $k_{p}$ and the least sensitive parameter being $k_{r}$. This partially explains why there is not much difference between the estimated values of $k_{r}$ across nutrient profiles. On the other hand, as pointed out in the model formulation, parameter unidentifiability maybe an issue for $k_{r},k_{p}$, and $k_{el}$. Moreover, the difference in sensitivity becomes clearer under higher uncertainty, but the overall sensitivity order remains the same. This is surprising since the elongation rate, which directly impacts the growth rate, is intuitively thought to be the most important parameter that governs the process. A possible explanation is that the impact of $k_{p}$ is amplified artificially due to the assumption of independence between $k_{p}$ and $k_{el}$. In our general formulation, since $k_{p}$ is proportional to $k_{el}$, the sensitivity of $k_{el}$ should increase. Alternatively, if we consider translation as a transport problem (e.g., cars moving along a single highway), then $k_{p}$ represents the high traffic point (or the bottle neck) of the system. Hence, $k_{p}$ controls the flow of the system. This effect is further amplified by the assumption that there is no aborting events after initiation. A simple way to address this is to simply allow $k_{p}$ to be a function of $k_{el}$. In other words, we can replace $k_{p}$ with a function of $k_{el}$ and a predetermined length of the initiating region on the mRNA strand. In addition, we may consider having a low but equal aborting rate after initiation. By doing so, we expect $k_{el}$ to be the most significant parameter in the general framework.

**Ribosomal dynamics under C- and P-limitation occur on a faster time scale than under of N-limitation.** Nitrogen contributes in many aspects of cell growth, such as in the production of proteins, DNA, and RNA. Using the values obtained from our estimations, we simulated the ribosomal dynamics under C-, N-, and P-limitation, see Fig. 5. We found that different types of nutrient limitation not only require cells to use different coping mechanisms, but also change the time scale of cell response. Under C- and P-limitation, we observed ribosomes reaching close to equilibrium (within 10%) significantly faster than under N-limitation for all three factions of the ribosomes. This may be due to the slow elongation rate under N-limitation, but other mechanisms may also play a role. We speculate that our observation is due to the involvement of *N* in forming macromolecules that are necessary in cell growth. Alternatively, since carbon is also present in these macromolecules in addition to energy pathways, and ATP concentrations in N-limited organisms are also high, our observation may also be an effect of energy limitation in the C- and P-limited treatments in that they reach equilibrium faster due to coupled energy and elemental limitation for ribosome synthesis. Unfortunately, we would need a higher resolution model, particularly with transcription, to study this hypothesis in more detail.

**Connection to the growth rate hypothesis.** We connected the Li et al. model with the framework by Loladze and Elser [31] to estimate the N:P ratio under the three nutrient limitation conditions. This is possible because both models share similar underlying processes and are intrinsically globally stable. Our results (Table 4) show consistent findings between experimental estimates and numerical estimates of the N:P ratios. We observed that the N:P ratios under P-limited are higher than under N-limited conditions at the same growth rate, which is expected. However, the Li et al. model does not contain the description of the transcription process, so we used the estimates from the study by Loladze and Elser for the transcription parameters (fraction of actively transcribing rRNA, $\phi_{r}$, and the transcription rate, $\sigma_{r}$). This may explain the similar N:P ratios under C-limited and N-limited conditions. Furthermore, $\sigma_{r}$ maybe smaller under P-limited condition, which would lead to a higher N:P ratio.

**Comparison of estimated parameters with literature**

1. **The translation abortion rate (**$k_{r}$**)**: previous studies suggest that it is possible for ribosomes to dissociate at equal rates at every codon (even after the initiating phase). By using an exponential decay model, the drop-off rate was estimated to be on the order of $10^{-4}$ to $10^{-2}$ per elongation step in stress-free environments [21], [22]. Our estimated range for $k_{r}$ falls within this range and further shows that aborting events occur at a relatively constant rate for similar growth rates. However, it is difficult to accurately isolate the aborting rate using a simple exponential decay model because the exponential drop-off observed in the ribosome density along the mRNA is due to multiple factors. Thus, a single-parameter model cannot distinguish them. If data permit, a direct extension to the model would be to examine the possibility of aborting events taking place after initiation.
2. **The translation elongation rate (**$k_{el}$**)**: our estimated values for the translation elongation rates agree with the experimentally derived values from the original study by Li and colleagues. Furthermore, our estimation also falls within existing estimated range for elongation rate 6–20 aa/sec [20], [48], [49], [50]. When comparing to similar experimental settings, our estimates also agree with the estimates by Iyer et al. for C and N starvation *E. coli* of about 12.9 aa/sec [13]. Additionally, the hopping rate is often set around 10 aa/sec in TASEP models. [19].
3. **The translation initiation rate (**$k_{f}$**)**: previous studies using the TASEP modeling framework and experimental data for *E. coli* growth estimated the ribosome-dependent initiation rate to be around 0.09–0.15 per second [17], [19]. This value is equivalent to $k_{f}R_{u}$ in our model, which works out to be approximately 0.11, 0.097, 0.19 per second under C-, N-, P-limited conditions, respectively, at equilibrium. This means our estimates for the initiation rates are within range of previous estimates.
4. **The transition rate into working ribosomes (**$k_{p}$**)**: is a unique parameter specific to our model, so it is difficult to find comparable estimates. On the other hand, we note that it is likely to depend on the length of the initiating of mRNA strand, the elongation rate, and the aborting rate. In future iteration, it is advisable to replace $k_{p}$ with a function of the elongation rate to eliminate this uncertainty.

There is significant value in the ability to predict cell growth based mainly on information about nutrient supply conditions. Being able to do so would allow us to naturally link supply to demand to biological function. For example, if we can make predictions about what the C:N:P ratios should be across different nutrient limitation treatments for a diverse group of organisms accurately, we can theoretically determine the growth rates off of elemental measurements of any animals out of the field. As there is currently no reliable way to carry out such task, it is difficult to extend these fundamental relationships governing growth on organismal scales to population and ecosystem-level dynamics. To this end, mathematical models are useful tools to facilitate this possibility. However, to do so, models need to be able to capture the expected qualitative behaviors and satisfy the standard biological constraints of the system. In this study, we provide a generalized framework for the translation process and study the mathematical and biological properties of the model. For simplicity, our modeling approach must make certain assumptions such as the independence between the translation and transcription process. Furthermore, transcription and translation processes are energy intensive, linked by the molecule Adenosine Triphosphate (ATP). Cells require energy to synthesize protein and DNA, which occur when energy is available via ATP reactions that themselves use phosphorus [51]. Thus, direct extension of the model may include explicit incorporation of the nutrient supply conditions and the coupling of transcription and translation processes with energy [52].

With regards to an extension to incorporate energy, the study by Scott et al. [4] inspires a simple possibility. Since cells must partition their energy usages into multiple processes, we can consider several compartments, each related to a major process such as transcription and translation elongation rates. For example, a compartment $A_{p}(t)$ can be used to keep track of the amount of energy used for translation. Then, $A_{p}(t)$ must influence the value of $k_{el}(\cdot )$, so we can introduce a new variable for the translation elongation rate that is a function of $k_{el}$ and $A_{p}$, perhaps $K_{el}(k_{el},A_{p})$. At equilibrium, we can argue that the allocation strategy is optimal, so an equilibrium must be reached, which should also be proven mathematically. This allows for a simplification of all the energy compartments into constants that represent their relative fraction of the total energy at equilibrium. Then the effect of the energy allocation strategy can be studied by estimating the values of these constants using energy data. If the optimal condition is loosened for generality, we would instead arrive at a much more complex problem of resource allocation, where cells are able to adjust their resource allocation continuously with respect to the differential changes in the environment for optimal growth. On the other hand, such system may be able to accurately reveal the different strategies that cells use in response to different growth condition.

Mathematically, certain interaction forms may provide interesting nonlinear dynamical behaviors for the model that contains biological insights [53]. For example, a more biologically realistic functional form of $f(R_{u},M_{f})$ may result in multiple equilibria within the biologically bounded region, or none at all. This may have significant implications for the biological system and help deepen our qualitative and quantitative understanding of the growth process.

## Declarations

### Author contribution statement

Tin Phan: Conceived and designed the experiments; Performed the experiments; Analyzed and interpreted the data; Contributed reagents, materials, analysis tools or data; Wrote the paper.

Changhan He: Performed the experiments; Analyzed and interpreted the data; Wrote the paper.

Irakli Loladze: Conceived and designed the experiments; Analyzed and interpreted the data; Wrote the paper.

Clay Prater: Analyzed and interpreted the data; Wrote the paper.

Jim Elser, Yang Kuang: Conceived and designed the experiments; Analyzed and interpreted the data; Contributed reagents, materials, analysis tools or data; Wrote the paper.

### Funding statement

YK, CH, IL and TP were supported by 10.13039/100000001National Science Foundation and 10.13039/100000002National Institutes of Health [DEB-1930728 and R01GM131405-02]. JJE was supported by 10.13039/100000001National Science Foundation [DEB-1930816] and CP was supported by 10.13039/100000001National Science Foundation [DEB-1930736].

### Data availability statement

Data included in article/supplementary material/referenced in article.

### Declaration of interests statement

The authors declare no conflict of interest.

### Additional information

No additional information is available for this paper.

## Appendix A

### A.1 Mathematical analysis

Theorem 1 Positivity
*The system is positive given positive initials.*

Proof Grouping terms and note that $R_{t}=R_{w}+R_{i}+R_{u}$ so $R_{t}>R_{w}$:

(23) $$\frac{dR_{i}}{dt}=\underset{>0}{\underset{︸}{k_{f}M_{t}(R_{t}-R_{w})+k_{f}R_{i}^{2}}}-\underset{>0}{\underset{︸}{\left(k_{f}M_{t}+k_{r}+k_{p}+k_{f}R_{t}+k_{f}R_{w}\right)}}R_{i},$$

(24) $$\frac{dR_{w}}{dt}=k_{p}R_{i}-\frac{k_{el}}{N_{aa}}R_{w}.$$

Since the negative terms for the rate variables are proportional to the variables, positivity is guaranteed with positive initials. □

Theorem 2 Boundedness
*The system was constructed under the assumption that* $R_{u}(t)+R_{i}(t)+R_{w}(t)=R_{t}=constant$ *and* $M_{f}(t)+M_{b}(t)=M_{t}=constant$ *for all* t≥0 *. We will verify that*

1. $\frac{dR_{t}}{dt}\equiv 0$ *,*
2. $\frac{dM_{t}}{dt}\equiv 0$ *.*

*Combine with positivity and bounded initials, this shows each state is bounded above by either* $R_{t}$ *or* $M_{t}$ *.*

Proof Separating the two systems for ribosomes and mRNA. **Ribosomes:** The system for ribosomes alone takes the form:

(25) $$\frac{dR_{u}}{dt}=k_{r}R_{i}+\frac{k_{el}}{N_{aa}}R_{w}-k_{f}M_{f}R_{u},$$

(26) $$\frac{dR_{i}}{dt}=k_{f}M_{f}R_{u}-k_{r}R_{i}-k_{p}R_{i},$$

(27) $$\frac{dR_{w}}{dt}=k_{p}R_{i}-\frac{k_{el}}{N_{aa}}R_{w}.$$

Adding together,

(28) $$\frac{dR_{t}}{dt}=\frac{dR_{u}}{dt}+\frac{dR_{i}}{dt}+\frac{dR_{w}}{dt}=0.$$

**mRNA:** The system for mRNA alone takes the form:

(29) $$\frac{dM_{f}}{dt}=k_{p}R_{i}+k_{r}R_{i}-k_{f}M_{f}R_{u},$$

(30) $$\frac{dM_{b}}{dt}=k_{f}M_{f}R_{u}-k_{r}R_{i}-k_{p}R_{i}.$$

Adding together,

(31) $$\frac{dM_{t}}{dt}=\frac{dM_{f}}{dt}+\frac{dM_{b}}{dt}=0.$$

 □

Theorem 3 Positive equilibrium
*With the biological bounds of* $R_{t}$ *and* $M_{t}$ *, then there is a unique positive equilibrium at* $\left(R_{i^{-}}^{⁎},R_{w^{-}}^{⁎}\right)$ *, where* $R_{i^{-}}^{⁎}=\frac{1}{A}\left(-B-\sqrt{B^{2}-4AC}\right)$ *and* $R_{w^{-}}^{⁎}=N_{aa}\frac{k_{p}}{k_{el}}R_{i^{-}}^{⁎}$ *.*

Proof Recall the system:

(32) $$\frac{dR_{i}}{dt}=k_{f}M_{t}R_{t}-k_{f}M_{t}R_{w}-(k_{f}M_{t}+k_{r}+k_{p}+k_{f}R_{t})R_{i}+k_{f}R_{i}^{2}-k_{f}R_{i}R_{w},$$

(33) $$\frac{dR_{w}}{dt}=k_{p}R_{i}-\frac{k_{el}}{N_{aa}}R_{w}.$$

Setting $\frac{R_{w}}{dt}=0$ and solving for $R_{w}^{⁎}$,

(34) $$R_{w}^{⁎}=N_{aa}\frac{k_{p}}{k_{el}}R_{i}^{⁎}.$$

Setting $\frac{dR_{i}}{dt}=0$, replacing $R_{w}^{⁎}=N_{aa}\frac{k_{p}}{k_{el}}R_{i}^{⁎}$ and organizing terms

(35) $$\underset{A}{\underset{︸}{k_{f}\left(1-N_{aa}\frac{k_{p}}{k_{el}}\right)}}{\left(R_{i}^{⁎}\right)}^{2}\underset{B}{\underset{︸}{-\left(k_{f}M_{t}N_{aa}\frac{k_{p}}{k_{el}}+k_{f}M_{t}+k_{r}+k_{p}+k_{f}R_{t}\right)}}R_{i}^{⁎}+\underset{C}{\underset{︸}{k_{f}M_{t}R_{t}}}=0$$

From the quadratic formula,

(36) $$R_{i^{\pm }}^{⁎}=\frac{1}{2A}\left(-B\pm \sqrt{B^{2}-4AC}\right).$$

Observe that

(37) $$B^{2}-4AC={\left(k_{f}M_{t}N_{aa}\frac{k_{p}}{k_{el}}+k_{f}M_{t}+k_{r}+k_{p}+k_{f}R_{t}\right)}^{2}-4\left(1-N_{aa}\frac{k_{p}}{k_{el}}\right)k_{f}^{2}M_{t}R_{t},$$

(38) $$>{\left(k_{f}M_{t}N_{aa}\frac{k_{p}}{k_{el}}+k_{f}M_{t}+k_{r}+k_{p}+k_{f}R_{t}\right)}^{2}-4k_{f}^{2}M_{t}R_{t},$$

(39) $$=2k_{f}^{2}M_{t}R_{t}+{\left(k_{f}M_{t}\right)}^{2}+{\left(k_{f}R_{t}\right)}^{2}+\underset{>0}{\underset{︸}{...}}-4k_{f}^{2}M_{t}R_{t},$$

(40) $$={\left(k_{f}M_{t}-k_{f}R_{t}\right)}^{2}+\underset{>0}{\underset{︸}{...}}>0.$$

This means there exists two real roots, or $\left(R_{i^{-}}^{⁎},R_{w^{-}}^{⁎}\right)$ and $\left(R_{i^{+}}^{⁎},R_{w^{+}}^{⁎}\right)$. If $1-N_{aa}\frac{k_{p}}{k_{el}}<0$ (or A<0), then $\sqrt{B^{2}-4AC}>|B|$, so $R_{i}^{⁎}$ is only positive if $R_{i}^{⁎}=R_{i^{-}}^{⁎}$. Otherwise if $1-N_{aa}\frac{k_{p}}{k_{el}}>0$ (or A>0), then both roots are positive. However, we see that $R_{i^{+}}^{⁎}=R_{t}/(1-N_{aa}\frac{k_{p}}{k_{el}})+\text{ positive terms }>R_{t}$. Thus, only $R_{i}^{⁎}=R_{i^{-}}^{⁎}$ is biologically realistic. Furthermore, if $1-N_{aa}\frac{k_{p}}{k_{el}}=0$ (or A=0), then $R_{i}^{⁎}=-C/B>0$. □

Theorem 4 Local stability
*The biological equilibrium at* $\left(R_{i^{-}}^{⁎},R_{w^{-}}^{⁎}\right)$ *is locally asymptotically stable (without additional conditions).*

Proof The Jacobian takes the form:

(41) $$J(R_{i}^{⁎},R_{w}^{⁎})=\left(\begin{array}{cc} -(k_{f}M_{t}+k_{r}+k_{p}+k_{f}R_{t})+2k_{f}R_{i}^{⁎}-k_{f}R_{w}^{⁎} & -k_{f}M_{t}-k_{f}R_{i}^{⁎}\\ k_{p} & -\frac{k_{el}}{N_{aa}} \end{array}\right).$$

Recall that $M_{t}=M_{f}+M_{b}=M_{f}+R_{i}$ and $R_{t}=R_{u}+R_{i}+R_{w}$, so $R_{i}<M_{t}$ and $R_{i}<R_{t}$. Now, consider the trace and determinant of the Jacobian matrix.

(42) $$T=2k_{f}R_{i}^{⁎}-k_{f}R_{w}^{⁎}-\frac{k_{el}}{N_{aa}}-(k_{f}M_{t}+k_{r}+k_{p}+k_{f}R_{t})$$

(43) $$=-k_{f}\underset{>0}{\underset{︸}{\left(M_{t}+R_{t}-2R_{i}^{⁎}\right)}}-k_{f}R_{w}^{⁎}-(k_{r}+k_{p})-\frac{k_{el}}{N_{aa}}<0.$$

(44) $$D=\frac{k_{el}}{N_{aa}}\left((k_{f}M_{t}+k_{r}+k_{p}+k_{f}R_{t})+k_{f}R_{w}^{⁎}-2k_{f}R_{i}^{⁎}\right)+k_{p}(k_{f}M_{t}+k_{f}R_{i}^{⁎})$$

(45) $$=\frac{k_{el}}{N_{aa}}\left((k_{r}+k_{p})+k_{f}R_{w}^{⁎}+k_{f}\underset{>0}{\underset{︸}{\left(M_{t}+R_{t}-2R_{i}^{⁎}\right)}}\right)+k_{p}(k_{f}M_{t}+k_{f}R_{i}^{⁎})>0.$$

Thus, $\left(R_{i}^{⁎},R_{w}^{⁎}\right)$ is locally asymptotically stable. □

Theorem 5 Global stability
*The biological equilibrium at* $\left(R_{i^{-}}^{⁎},R_{w^{-}}^{⁎}\right)$ *is globally asymptotically stable in the region bounded by*

$$C=\{R_{i},R_{w}>0;R_{i}+R_{w}\leq R_{t};R_{i}\leq M_{t}\}.$$

Proof First, we use Bendixson-Dulac criterion to show that there is no non-trivial orbit in the biological region bounded by *C*. Let $\phi (R_{i},R_{w})=\frac{1}{R_{i}}$. Consider

(46) $$\Omega =\frac{\partial }{\partial R_{i}}\left[\frac{1}{R_{i}}\left(k_{f}M_{t}R_{t}-k_{f}M_{t}R_{w}-(k_{f}M_{t}+k_{r}+k_{p}+k_{f}R_{t})R_{i}+k_{f}R_{i}^{2}-k_{f}R_{i}R_{w}\right)\right]\;+\frac{\partial }{\partial R_{w}}\left[\frac{1}{R_{i}}\left(k_{p}R_{i}-\frac{k_{el}}{N_{aa}}R_{w}\right)\right]=\frac{\partial }{\partial R_{i}}\left[\frac{k_{f}M_{t}R_{t}}{R_{i}}-\frac{k_{f}M_{t}R_{w}}{R_{i}}-(k_{f}M_{t}+k_{r}+k_{p}+k_{f}R_{t})+k_{f}R_{i}-k_{f}R_{w}\right]$$

(47) $$\;+\frac{\partial }{\partial R_{w}}\left[k_{p}-\frac{k_{el}}{N_{aa}}\frac{R_{w}}{R_{i}}\right]$$

(48) $$=\left(-\frac{k_{f}M_{t}R_{t}}{R_{i}^{2}}+\frac{k_{f}M_{t}R_{w}}{R_{i}^{2}}+k_{f}\right)-\frac{k_{el}}{N_{aa}}\frac{1}{R_{i}}$$

(49) $$=\frac{k_{f}}{R_{i}^{2}}\left[\left(M_{t}R_{w}-M_{t}R_{t}+R_{i}^{2}\right)-\frac{R_{i}}{k_{f}}\frac{k_{el}}{N_{aa}}\right].$$

Now, by the model assumption, $R_{i}=M_{b}$. Furthermore, the boundedness and positivity of the system in *C* imply $M_{b}<M_{t}$ and $R_{i}+R_{w}<R_{t}$, so we can make the following comparison

(50) $$\Omega =\frac{k_{f}}{R_{i}^{2}}\left[\left(M_{t}R_{w}-M_{t}R_{t}+M_{b}R_{i}\right)-\frac{R_{i}}{k_{f}}\frac{k_{el}}{N_{aa}}\right]$$

(51) $$<\frac{k_{f}}{R_{i}^{2}}\left[\left(M_{t}R_{w}-M_{t}R_{t}+M_{t}R_{i}\right)-\frac{R_{i}}{k_{f}}\frac{k_{el}}{N_{aa}}\right]$$

(52) $$<\frac{k_{f}}{R_{i}^{2}}\left[M_{t}\underset{<0}{\underset{︸}{\left(R_{w}+R_{i}-R_{t}\right)}}-\frac{R_{i}}{k_{f}}\frac{k_{el}}{N_{aa}}\right]$$

(53) <0.

Thus, the system does not have a periodic orbit. Therefore, the unique positive steady state is globally asymptotically stable by Poincaré-Bendixson theorem. □

### A.2 Convergence and sensitivity analyses

In order to test the convergence result and the sensitivity of the ribosomal dynamics under different initial conditions, we carry out simulations under four different scenarios: (1) $R_{u}=0.9R_{t},R_{i}=0.05R_{t},R_{w}=0.05R_{t}$; (2) $R_{u}=0.8R_{t},R_{i}=0.10R_{t},R_{w}=0.10R_{t}$; (3) $R_{u}=0,R_{i}=R_{t},R_{w}=0.0$; (4) $R_{u}=0,R_{i}=0,R_{w}=R_{t}$. The results are presented in Figure 7, Figure 8, Figure 9, which agree with our results in Figure 3, Figure 4, Figure 5.
